# Ex Vivo and Simulation Comparison of Leakage in End-to-End Versus End-to-Side Anastomosed Porcine Large Intestine

**DOI:** 10.3390/bioengineering12070676

**Published:** 2025-06-20

**Authors:** Youssef Fahmy, Mohamed Trabia, Brian Ward, Lucas Gallup, Whitney Elks

**Affiliations:** 1 Department of Mechanical Engineering, Howard R. Hughes College of Engineering, University of Nevada, Las Vegas, NV 89154, USA; fahmy@unlv.nevada.edu (Y.F.); gallup@unlv.nevada.edu (L.G.); 2 Department of Surgery, Kirk Kerkorian School of Medicine, University of Nevada, Las Vegas, NV 89106, USA; brian.ward@unlv.edu (B.W.); whitney.elks@unlv.edu (W.E.)

**Keywords:** colorectal anastomosis, end-to-end, end-to-side, anastomotic leakage, simulation of stapled anastomoses

## Abstract

Anastomotic leaks after colorectal resection are serious surgical complications. We have compared the integrity of two common colorectal anastomosis techniques, end-to-side (ES) and end-to-end (EE), to control specimens using a novel experimental setup that mimics anastomotic air leak tests, which are typically performed during surgeries. Freshly harvested porcine colonic sections from 23 F1 cross-species pigs were used. Pressure measurements and video imaging were used to monitor the ex vivo experiments on EE, ES, and Control specimens. Using EE (*n* = 16), ES (*n* = 12), and Control (*n* = 22) specimens, leak pressure was 282.6 ± 3.0 mm Hg for EE, 282.8 ± 2.6 mm Hg for ES, and 294.4 ± 12.1 for the Control. Time to leakage was 106.3 ± 28.1 s for EE, 263.9 ± 2127.0 s for ES, and 194.5 ± 90.2 s for the Control. We found that, while EE and ES have nearly identical leak pressures, ES was superior in terms of time to leakage and tissue expansion, which may explain why ES anastomoses have a lower clinical anastomotic leak rate. Two dependent variables representing stress and strain of colonic tissues were introduced. These variables showed ES was comparable to the Control. The experiments were simulated successfully using the finite element method (FEM). This research provides a reproducible ex vivo system with a corresponding FEM system to study the differences between anastomosis techniques and may help design anastomoses with lower leak rates and improve patient outcomes in colorectal surgeries.

## 1. Introduction

**Figure 1:**
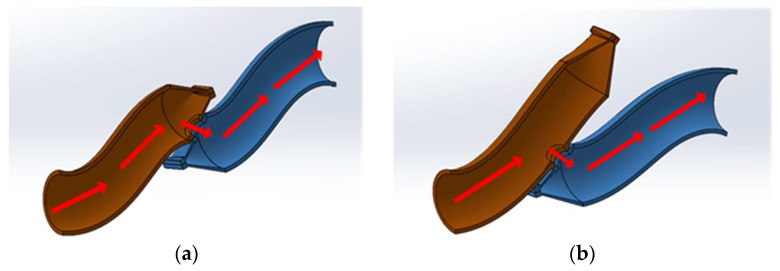
Two possible approaches for stapled colorectal anastomosis: (**a**) end-to-end (EE), (**b**) end-to-side (ES).

A colorectal anastomosis is a surgical procedure that reattaches the colon after a part of it has been removed. Colorectal anastomosis has been a standard treatment for multiple gastrointestinal conditions, including colorectal cancer. The surgery entails a partial colectomy to remove a part of the colorectal tract, followed by reattaching the remaining sections using several approaches, including sutures and stapling. Multiple techniques have been proposed and implemented, leading to reduced complications and enhanced patient recovery [1]. In particular, circular double staplers have increasingly been used, as they are repeatable and can decrease the duration of the surgery and the potential for abdominal contamination [2]. Two commonly used stapling techniques are end-to-end and end-to-side. The main difference between these two techniques is in the distance to the attachment of the distal and proximal ends of the reconstructed colon. Both techniques start with sealing the openings of the tract using linear staples, followed by circular staples to form the anastomosis. In EE anastomosis, a circular staple line occurs close to the linear staple line, while in ES anastomosis, the circular staples are placed farther away from the linear staple line, creating a pocket on the proximal end of the circular staples. Figure 1 shows the schematics of these two approaches.

An important step in colorectal anastomosis is reconnecting the colorectal tracts securely [2] to avoid anastomotic leakage (AL), which is a major surgical complication of colorectal anastomoses with reported leaks varying from about 2 to 30% of patients [2,3,4,5]. It was estimated that AL resulted in USD 28.6 million in additional costs for every 1000 patients undergoing colorectal surgery [6], with the average cost of a leak exceeding USD 50,000 [7]. AL is partially related to the strength of a freshly completed bowel anastomosis with leakage pressure commonly used to assess the quality of a fresh intestinal suture or staple line [8]. This test is sometimes labeled burst testing in literature. Surgeons typically test the integrity of an anastomosis by pumping pressurized air through the colon with a colonoscope immediately after creating the anastomosis and monitoring evidence of leakage by looking for bubbles from the saline submerged anastomosis [9,10].

Despite the recent advances in colorectal anastomosis surgeries, AL is not fully understood [11]. The following is a brief overview of research comparing various anastomotic techniques. This review is divided by the nature of research: randomized clinical trials, in vivo studies, and ex vivo studies. Randomized clinical trials can be effective in understanding long-term outcomes of colorectal anastomosis, including issues such as morbidity, the clinical leakage rate, and mortality. In one study, 615 colorectal resections with stapled anastomoses were evaluated. It was found that anastomotic leakage occurred in 1.5% of all patients [12]. A systematic review of multiple databases comparing stapled and sutured colorectal anastomoses was conducted. It was concluded that the two techniques were equivalent [13]. Clinical and experimental articles on colorectal anastomotic techniques and healing were searched in terms of suture material and format, single- and double-layer sutures, interrupted and continuous sutures, and hand-sewn versus stapled and compression colorectal anastomosis. The results show that single-layer continuous sutures were preferable; however, this type of suturing suffers the nonstandard nature of the technique compared to stapled and compression colorectal anastomoses [14]. The risk of AL was assessed for more than 70,000 patients who underwent elective low anterior resection with an AL rate of about 14% [5]. In another study, colorectal anastomosis techniques were assessed based on complications such as leakage, stricture, and bleeding, using 684 cases in a single hospital. Three techniques were considered: hand-sewn, end-to-end, and triangulating anastomosis. The results indicate that leakage was significantly lower in laparoscopic surgeries and triangulation [15]. A study evaluated the complications associated with ileocolic anastomoses performed using stapling and handsewn techniques using multiple medical databases. It was concluded that stapled end-to-end anastomoses are associated with fewer leaks than handsewn anastomoses [16]. The outcome for patients who underwent end-to-end and end-to-side anastomoses after laparoscopic resection was assessed, and end-to-end anastomosis yielded lower rates of severe complications and reintervention in low–mid rectal anastomoses [17]. However, in another study, articles from various databases that presented randomized controlled trials of end-to-end and end-to-side anastomoses were compared. The primary outcome was the anastomotic leak rate. The results showed that end-to-side anastomosis significantly reduced the risk of anastomotic leak after low anterior resection [18]. The risk of AL between manual circular staplers with two rows versus three rows was compared, and the risk of AL in both staplers was found to be similar [19]. However, a recent study has concluded that three-row staples may reduce the risk of anastomotic leak in left-sided colorectal anastomoses [20].

In vivo experiments can lead to a better understanding of the immediate effects of colorectal anastomosis. The validity of the burst strength test of anastomoses in various animal experiments was assessed. It was concluded that the burst strength test is a meaningful parameter for evaluating AL [21]. A study comparing canine colorectal anastomotic burst pressure was conducted using a biofragmentable anastomotic ring and stapled and sutured colon anastomoses that were sequentially placed in each of the 48 dogs. The results indicate that the ring anastomoses had the greatest strength on the day of surgery, sutured anastomoses were the strongest on the third day, and all techniques were comparable by the seventh day [8]. A model to quantify the proper pressure in air-leak testing was explored using five pigs that underwent colonoscopic and bulb syringe insufflations [22]. While testing results were statistically similar, it was concluded that pressures should be in the order of 10 to 13 mm Hg. In vivo burst strength testing in a domestic pig model was conducted to compare sutures and staples. After 24 h of continuous negative pressure therapy, each anastomosis was subjected to burst strength testing. The results indicate that stapled anastomoses have significantly lower burst strength than sutured anastomoses [23].

Ex vivo experiments have the potential to clarify the integrity of anastomosis immediately after surgery. Additionally, ex vivo experiments are more controlled compared to those conducted in vivo, leading to a better comparison of the various techniques as well as the ability to simulate the experiments and test different anastomosis designs. A study using fresh human colon segments compared ten each: hand-sutured, biofragmentable anastomotic rings, and two-row stapled end-to-end anastomoses based on early burst pressure. The results showed no statistically significant differences between these three techniques [24]. A study to compare compression ring to end-to-end colorectal anastomosis techniques was conducted using 18 pigs. Six pigs were sacrificed immediately after the procedure while twelve pigs were sacrificed after two weeks for an ex vivo study. Leak pressures, circumferences, and leak rates were identified. The results indicated that compression anastomoses had higher mean leak pressures than stapled anastomoses immediately after the anastomosis. However, there was no difference between leak pressures [25]. An ex vivo experimental setup was developed for in vitro burst testing of an end-to-end porcine specimen. The specimen was submerged in water with the bottom end plugged while a colored liquid was pumped at the top end [26]. Using 15 pigs, five methods of colorectal anastomosis were performed on the porcine rectum and colon: single-stapled double-purse-string, double-stapling, side-to-side with a linear stapler, side-to-side with a circular stapler, and side-to-side with hand-sewn reinforcement (*n* = 6 for each method). Results showed that burst pressures were significantly higher in side-to-side with a linear stapler than in side-to-side with a circular stapler anastomosis [27]. A leakage test experiment was developed to test the integrity of end-to-end anastomoses. The anastomosis operations were conducted on six living pigs. These anastomoses were then cut, and an ex vivo burst test was performed to evaluate the maximum intraluminal pressure. Fluid was pumped into one end while the other was sealed using hemostatic forceps [28]. Four colonic anastomosis techniques (*n* = 4 for each method) were performed using canine cadavers: two types of hand-sewn and two types of circular stapled end-to-end colonic anastomoses. These techniques were evaluated based on leakage pressure. The results indicate that lower pressure leakage was associated with stapled anastomoses [29].

Review of the literature demonstrates that prior studies have examined anastomoses with very different experimental models and have yielded highly variable experimental outcomes. These results may be partially explained by the fact that in vivo AL studies depend on a multitude of factors including overall organism health and co-morbidities, perioperative care, blood flow to the anastomosis, and surgical technique; the isolated effect of physical anastomotic configurations on AL remains unclear. The aim of this research was to create a reproducible ex vivo experimental system to study the differences in the mechanical characteristics and leak pressures of two different anastomotic configurations, end-to-end and end-to-side. While most researchers have only reported leak pressure, we aimed to relate it to factors such as the dimensions of the colonic specimens at the onset of leakage to account for the variation in these dimensions. We also sought to model these two anastomotic configurations with finite element modeling. This deeper understanding may help design colorectal anastomoses with lower leak rates and improve patient outcomes.

## 2. Materials and Methods

### 2.1. Specimen Collection

This research was evaluated by the Institutional Animal Care and Use Committee at the University of Nevada, Las Vegas, who deemed it to be exempt since the institutional policies apply to live animal care and animals that are sacrificed for research purposes. The colorectal tissues used in this project would have been normally discarded, as the animals were sacrificed for food consumption.

### 2.2. Specimen Preparation

Freshly harvested porcine colon tracts were collected from 23 pigs of the F1 cross-species (a Yorkshire and Landrace mix) from a local farm (Las Vegas Livestock, LLC, Las Vegas, NV, USA). The population included male and female pigs whose ages ranged from 3 to 8 months. At this stage, the size of the porcine colon is comparable to its human counterpart despite their anatomical configuration differences. Additionally, the histologic structures of the porcine and human colonic tissues are comparable even though they are not identical as shown in [30]. This comparison may explain their widespread use in studies as noted in the previous section.

The entire intestinal tracts (jejunum to anus) were collected immediately after postmortem. The colon was separated from its mesentery to create straight segments of colon. The luminal contents of the colons were drained, and three adjacent colonic segments were harvested from a segment of each pig’s colon with roughly uniform diameters (approximately 35 mm). These segments were used to prepare three specimens: EE, ES, and Control. Specimens were kept in an insulated cooler during transportation. The time between harvesting and experiments ranged from three to four hours. We were able to extract more than three segments from some pigs, leading to 30 specimens of each type in total.

**Figure 2:**
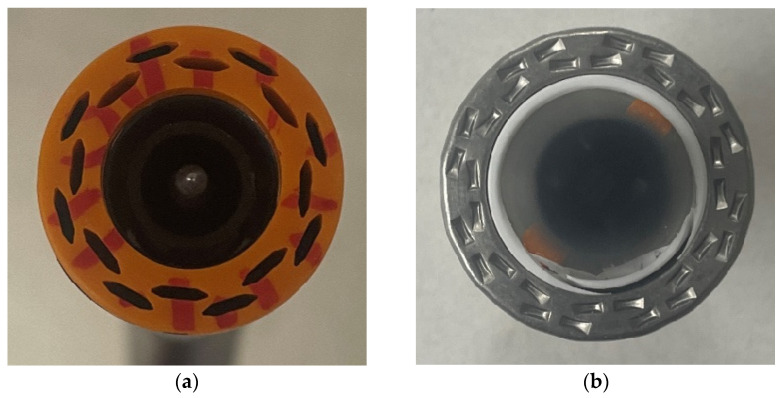
Circular staple: (**a**) launcher, (**b**) anvil.

Trained surgeons prepared the EE and ES anastomosis specimens. Colonic sections were connected using Ethicon PROXIMATE^®^ Regular Tissue Linear Cutter (Ethicon Inc., Raritan, NJ, USA), 75 mm [31]; and Ethicon^®^ Circular Stapler Curved (ILS), a 21 mm diameter stapler [32]. A total of 16 staples were spread equally between two concentric circles, with a difference in diameter of 0.20 mm between the outer and inner diameters. On both circles, staples were placed every 45° with a 22.5° overlap between the inner and outer circles; see Figure 2.

**Figure 3:**
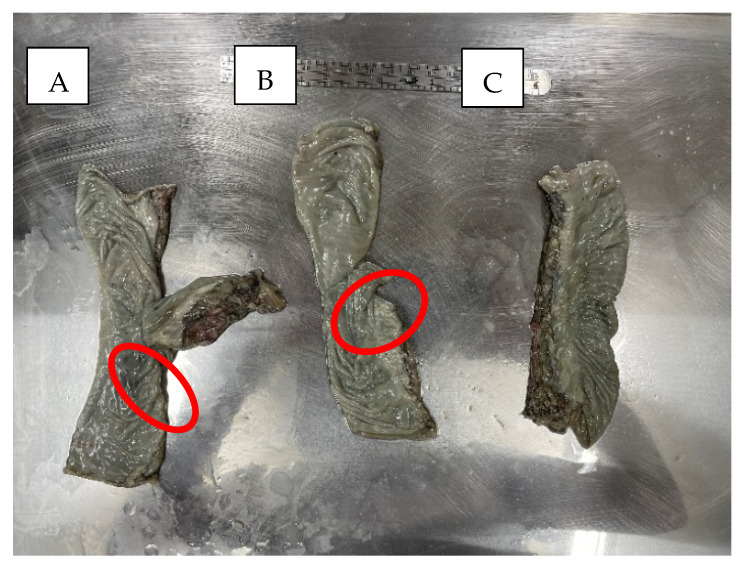
Prepared specimens, (A) ES, (B) EE, and (C) Control specimens. The locations of the circular stapled anastomoses are shown using red ellipses.

In the case of EE anastomosis, the stapler anvil was placed approximately 2 cm away from the linear staple lines of both the proximal and distal specimen ends to avoid overlapping of the staple lines while still having an end-to-end configuration when inflated. In the case of ES anastomosis, the same procedures were applied, except that the anvil in the proximal and distal ends would be placed 8 cm and 2 cm away from the linear staple line, respectively, which would produce a pouch on the proximal end of the ES anastomosed specimen. The circular staple line was placed centrally through the antimesenteric side of the colon for both EE and ES specimens. The Control specimens were used as a comparison baseline. These specimens were intact colonic sections of approximately the same length as the EE and ES stapled sections (13 cm). Figure 3 shows the three types of specimens. Using a digital Mitutoyo Caliper (Mitutoyo, Kawasaki, Japan) with 0.01 mm accuracy, two co-authors were tasked with measuring the thickness of the colorectal tissues. Both had experience from their earlier research where they measured the thickness of specimens used to determine the colorectal constitutive material model [33]. The tissue thickness of each specimen was measured three times at the top, middle, and bottom before testing. 

### 2.3. Anastomotic Leakage Experimental Setup

An ex vivo experimental setup was developed to identify the leak pressure of colorectal anastomoses. Specimens were air-inflated at a constant rate while underwater. AL was defined as the instant at which an anastomosis leaked, as indicated by the generation of air bubbles. Bubble formation is associated with a rapid drop in internal pressure. To closely simulate the in vivo surgical procedure, the specimens were fully submerged in a temperature-controlled water tank with temperatures set to 38 °C to mimic internal body temperature. Similarly, internal body pressure was simulated by submerging the specimens under approximately 100 mm of water, which corresponds to the abdominal pressure of 7.0 mm Hg [34].

Based on the limited available research on stapled colon anastomoses in porcine models, we made numerous assumptions to calculate the sample size needed to adequately power this study. Our assumptions were generally conservative to ensure the study would be adequately powered. AL pressure estimates in stapled anastomoses vary widely in literature but generally range from 20 to 120 mm Hg, as described in Section 1. Assuming our ES anastomosis group has a mean colon leak pressure of 80 mm Hg with a standard deviation of 30 mm Hg and our EE anastomosis group has a mean colon leak pressure of 60 mm Hg, each group requires 35 samples with an alpha value set at 0.05 and power set at 80%. We requested 50 circular and linear cutting staplers per arm to allow for stapler malfunction/technical error, optimization of study parameters, and several specimens for linear stress/strain parameter development. Variance analysis indicated failure to reject the null hypothesis at the 5% significance level for all variables except for time to leakage for EE versus Control specimens.

**Figure 4:**
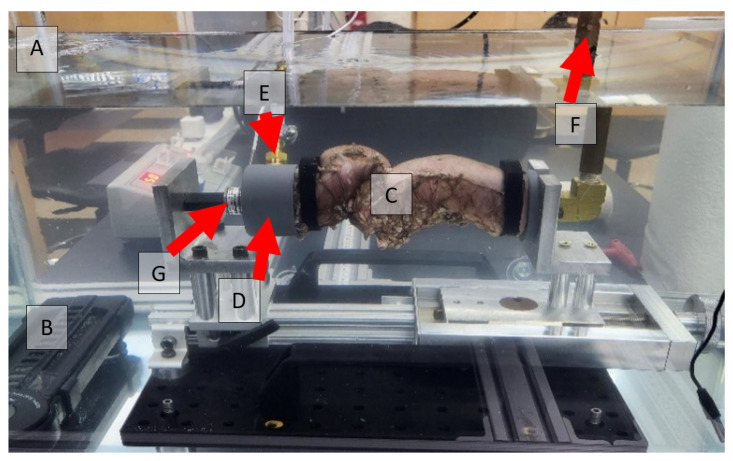
Experimental setup for leakage testing: (A) water tank, (B) temperature controller, (C) specimen, (D) adapter and clamp, (E) air input from pump, (F) pressure transducer, (G) load cell.

**Figure 5:**
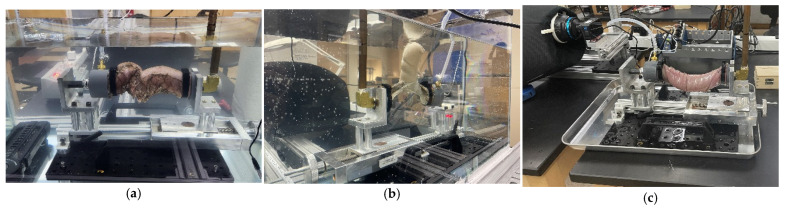
Examples of the three leakage testing groups: (**a**) EE, (**b**) ES, (**c**) Control (before moving it into the water tank).

The two unstapled distal and proximal ends of the colon sections were connected to inlet and outlet adapters using snap-grip hose clamps to create an air-tight seal without damaging the tissues. A peristaltic pump was used to inflate the anastomosis at a rate of 50 mL/min. A pressure transducer at the outlet monitored the pressure during the experiment. To ensure that the tissue was not stretched before the start of inflation, a load cell was placed behind the inlet adapter. Pressure and load data were collected at a rate of 30 samples per second. Pressure data were monitored to identify the instant at which the pressure started increasing, based on the increase in the slope of the pressure–time curve. Two Back-Bone Modified GoPro Hero 10^®^ cameras (GoPro, San Mateo, CA, USA), each with a Nikon AF-S Nikkor^®^ 18–140 mm lens (Tokyo, Japan), were placed on both sides of the tank to monitor the experiment. These cameras collect images at a rate of 60 frames per second with a 5.3 k resolution. Light cones were placed around the camera to the tank to reduce light reflection and improve the image quality. The experiment was assembled outside the water tank and then installed immediately after the setup was completed. Figure 4 shows the experimental setup while EE, ES, and Control specimens are presented in Figure 5.

**Figure 6:**
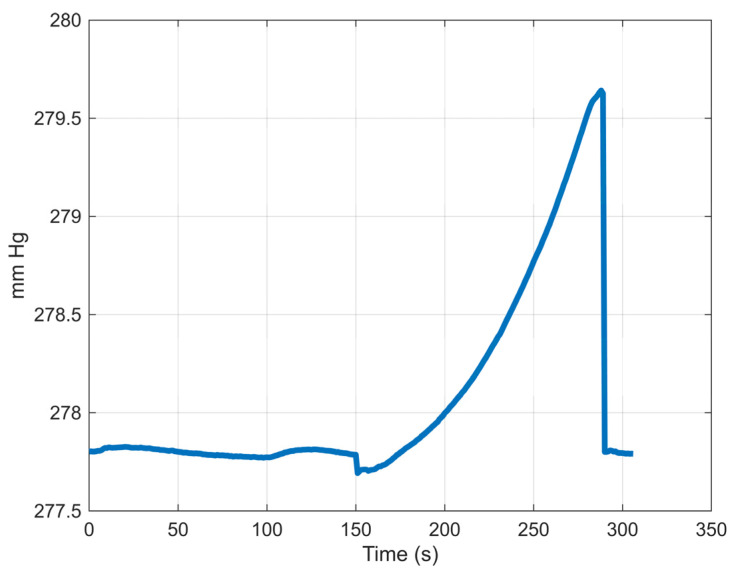
A typical colorectal anastomosis leak testing pressure–time history.

**Figure 7:**
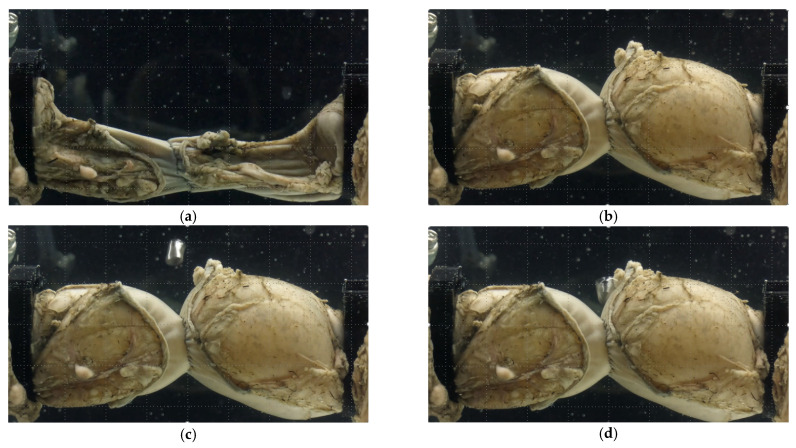
Phases of the leakage testing for an EE anastomosis: (**a**) colonic section before pumping air, (**b**) section just before the leakage initiation, (**c**) section at the onset of leakage, (**d**) section near the end of the test.

Figure 6 shows a typical pressure time history of an experiment. It may be useful to explain the phases of the testing. Originally, the colonic section was folded due to the pressure of the surrounding water and gravity forces. As the air was pumped, the colonic section assumes a cylindrical form. Time is recorded at this moment. However, the continued pumping of air and the hyperplastic nature of the tissues led to the section inflating at different rates. The anastomosis region experienced the least expansion due to the staple-imposed restrictions on this region. Once a puncture hole of a staple became elongated, air escaped, and we could notice bubble formation and a sudden drop in pressure. Since the air was pumped continuously during the experiment, the colonic section did not go back to the original shape while the pressure returned to close to its original value. The phases of the leakage are shown in Figure 7. In all three groups, time to leakage was measured from the instant the colorectal sections assumed cylindrical configuration to the moment of bubble formation.

### 2.4. Data Extraction and Processing

The appearance of bubbles during pressurization was always associated with a significant pressure drop. Based on synchronization of the video and pressure data, it was clear that maximum pressure was associated with leakage of the specimens. However, we did not stop an experiment based on monitoring the pressure. Instead, the decision was based on bubble formation.

**Figure 8:**
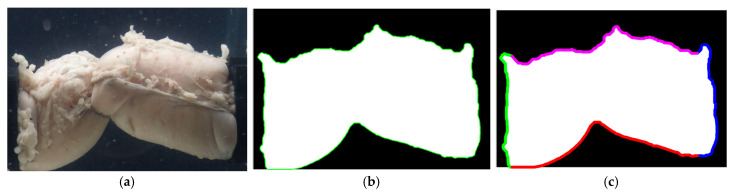
An example of EE image processing: (**a**) actual image, (**b**) corresponding black and white image, and (**c**) separation of the border into left, right, upper, and lower edges.

The image processing procedure is described in the remainder of this section. Based on pressure data, initial and final frames were identified. Each frame underwent the following processes: conversion of the image to gray scale, then into black and white. A custom Matlab^®^ R2024a (Natick, MA, USA) code identified the pixels that corresponded to the borders of the specimen. These pixels were then separated into four sets describing the two clamped left and right edges and the upper and lower edges of the specimen; see Figure 8. The average distance between the left and right edges was used to determine the length of the specimen. Similarly, the average distance between the upper and lower edges was used to measure the change in the diameter of the specimen throughout the experiment.

### 2.5. Statistical Analysis

Location differences within each specimen group were tested using paired *t*-tests, with a Bonferroni-adjusted significance level less than 0.0167 being required to reach significance at the *p* = 0.05 level. To further understand the results, multiple comparisons were conducted using Dunnett’s test.

### 2.6. Simulation of Anastomotic Leak Testing Experiments

Reliable simulation of anastomotic leakage can help understand this phenomenon and design better surgical techniques and stapling arrangements. Few researchers have developed simulations of anastomotic leakage [35]. For example, a finite element method (FEM) model was created to simulate the interaction of the tissues and the staples and to estimate leakage pressure of EE anastomosis [28]. Recently, a study was conducted to assess the effectiveness of matrix staple line reinforcement through a combination of experiments and FEM [36]. Progression of this research will use the FEM model to predict optimal anastomosis designs that can be confirmed with ex vivo experimentation.

**Figure 9:**
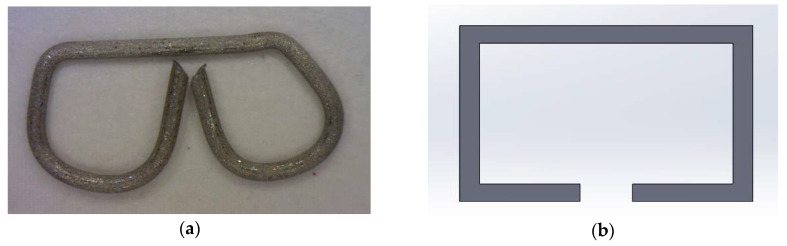
Single circular staple: (**a**) actual, (**b**) simplified model.

To further investigate this issue, finite element models were developed within ANSYS^®^ MECHANICAL 2021 R2 software (Canonsburg, PA, USA) to simulate EE, ES, and Control groups. It was assumed that the colonic sections are cylindrical as they are under pressure. The stapler bends the staples into a B-shape due to the anvil geometry, leading to the staple penetrating the tissues at four points. However, due to the complex interaction between the sharp edges of the staple and the soft colon tissue, a simplified staple model with a square cross-section of the same cross-sectional area as the original staple was created; see Figure 9. A bilinear titanium material model was used to model the nickel–titanium staple material; see Table 1.

**Figure 10:**
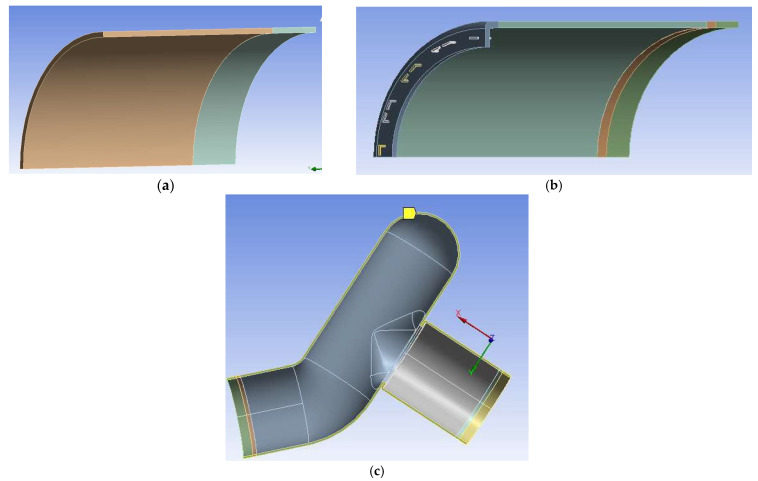
FEM geometries of (**a**) one-eighth Control, (**b**) one-eighth EE anastomosis, and (**c**) one-half ES anastomosis. Models are not shown on the same scale.

The geometries of the Control and EE anastomosis were presented using quarter and one-eighth models, respectively, while the ES anastomosis was represented using a half-model; see Figure 10. All the specimen groups were modeled using the average diameter and thickness. The smaller parts in these three figures correspond to the portion that was fixed around the adapter and clamp. The hole pattern of the staple was also modeled based on the pattern of the stapler launcher and anvil; see Figure 2. Contact between staples and neighboring tissues were defined using bonded contact between the inner surface of the staple and the top surface of the tissue. Frictionless contact was defined between the outer and side surfaces of the staple legs and the adjacent surfaces of the holes.

**Figure 11:**
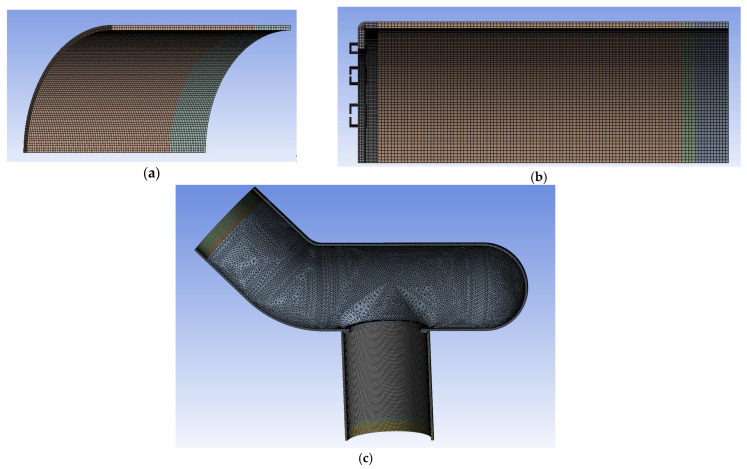
FEM meshes of (**a**) one-eighth Control, (**b**) one-eighth EE anastomosis, and (**c**) one-half ES anastomosis. Models are not shown on the same scale.

**Figure 12:**
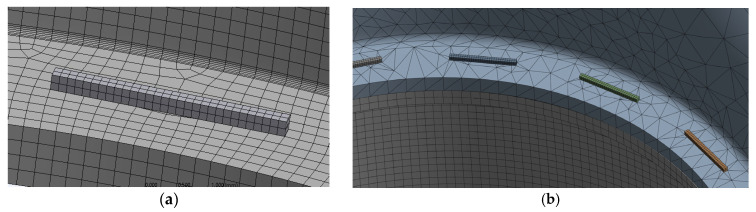
Detail of the meshes used for the staples and the surrounding tissues: (**a**) EE and (**b**) ES. Models are not shown on the same scale.

Using quadratic elements, a multi-zone meshing feature was used to generate hexahedral mesh with a smooth transition between the various parts of the models; see Figure 11. The region surrounding the staples was set to a finer mesh of 0.25 mm. Figure 12 shows a zoomed detail of the mesh used for staples and their surrounding region. Table 2 lists the final values used for the rest of this work. Element sizes were doubled and halved for the three models. These models were tested, showing that the meshes presented in Figure 11 are acceptable.

**Figure 13:**
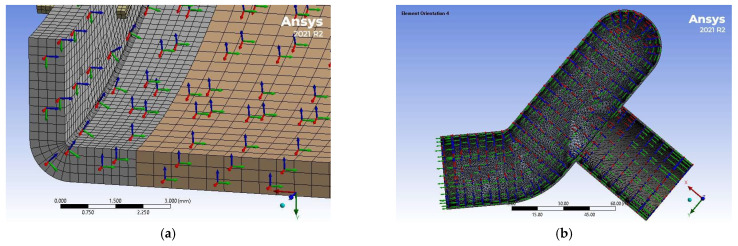
Using local frame elements to define the material model: (**a**) EE and (**b**) ES. Models are not shown to the same scale.

Colorectal tissues were modeled using a hyperelastic anisotropic constitutive model, which combines the Yeoh hyperplastic material model with two sets of anisotropic fibers that were oriented within the tissue [33]. This model was based on uniaxial testing in the circumferential and longitudinal directions of the freshly harvested porcine colorectal tissues. The parameters of the constitutive model, as outlined in Table 3, were input into ANSYS^®^ 2023 R1 using the Parametric Design Language (APDL) (Canonsburg, PA, USA). APDL is a structured scripting language used to interact with the Ansys Mechanical solver. The constitutive model was used for the tissues in the three models. Colorectal tissue material elements were oriented to match the axes of the material model. Therefore, the local *Z*-axis of each element was normal to the surface of the element while the *X*- and *Y*-axes were aligned with the circumferential and longitudinal *Y*-axes of the material model, respectively; see Figure 13.

Displacement boundary conditions were implemented to ensure that the symmetrical surfaces do not exceed these planes. The outer, inner, and end surfaces of the clamped part were also fixed. A ramp, starting at zero and ending at 210 mm Hg (2.8 × 10^−2^ MPa), represented the internal pressure applied to the inner surface of the three models.

## 3. Results

### 3.1. Experimental Results

**Figure 14:**
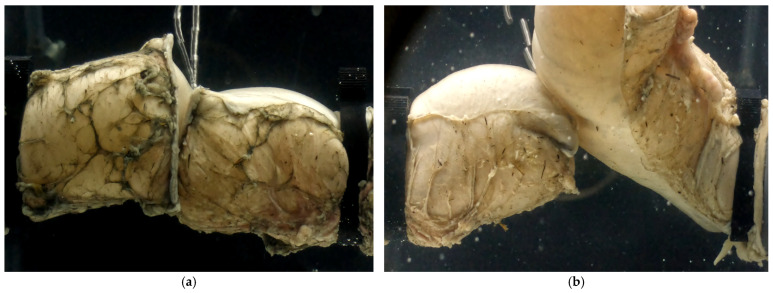
Bubble formation in AL testing: (**a**) EE and (**b**) ES.

Successful tests were those in which leakage occurred at the circular staple lines for EE and ES specimens or at the tissues for the Control specimens. Excluded specimens were those that had either end slip out of the clamps or those with corrupt or low-quality videos, which were either the result of low/bright lighting or, in a few cases, the camera being out of focus. A few EE and ES specimens were also excluded because they experienced failure of tissue away from the staples resulting in the formation of holes and premature bubble generation away from the anastomosis, likely from injury to the colon tissue during the specimen clamping process. These tissue failures were secondary to injury to the tissue during the clamping process and not relevant to the experimental question being asked. Figure 14 shows examples of bubble formation at the onset of AL in the case of EE and ES anastomoses, respectively. Table 4 summarizes the leakage testing based on the type of failure. In terms of this research, EE and ES circular staple leakage are the most relevant. These cases were compared with the tissue failure of the Control group.

**Figure 15:**
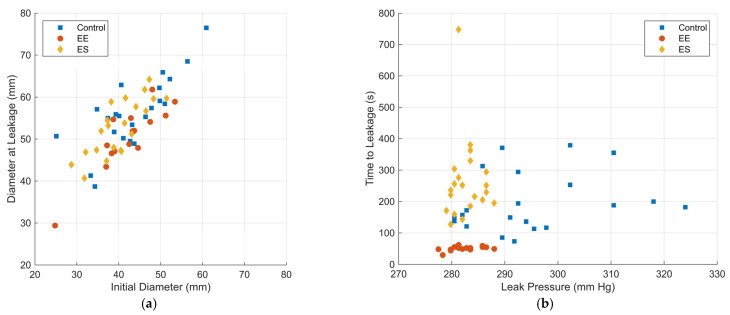
Raw data plots: (**a**) diameter at leakage versus initial diameter and (**b**) time to leakage versus leak pressure.

Raw data plots of the diameter at leakage versus the initial diameter and time to leakage versus leak pressure are shown in Figure 15. For statistical comparisons, the data were normally distributed. Mean and standard deviation values for all mechanical testing measures were calculated for each specimen group. Outlier data were identified using Chauvenet’s criteria and removed from datasets. All data are represented as mean ± SEM. Table 5 lists pre- and post-pressure dimensions of the specimens, leak pressure, and time to leakage for these three groups. Table 6 lists *p*-values and confidence intervals for these comparisons. Multiple comparisons using Dunnett’s test are shown in Table 7.

To better understand these experimental results, it may be possible to reduce the effect of variation in the colonic tract dimensions by combining the variables of Table 5 into two new variables, representing change in stress and strain. One variable, ∆σ, combined leak pressure with the difference between diameter at leakage and initial diameter and thickness. The other variable, ϵ, combined the change in diameter at leakage with the initial diameter. The following formulas describe these two variables:

(1) $$∆\sigma =\frac{P\left(D_{l}-D_{i}\right)}{t}$$

(2) $$\epsilon =\frac{D_{l}-D_{i}}{D_{i}}$$

where $D_{i}$ and $D_{l}$ are the initial diameter and diameter at leakage, respectively, t is the tissue thickness, and P is the leak pressure. Table 8 lists the average and standard deviation of these two variables for the three specimen groups.

### 3.2. Simulation Results

**Figure 16:**
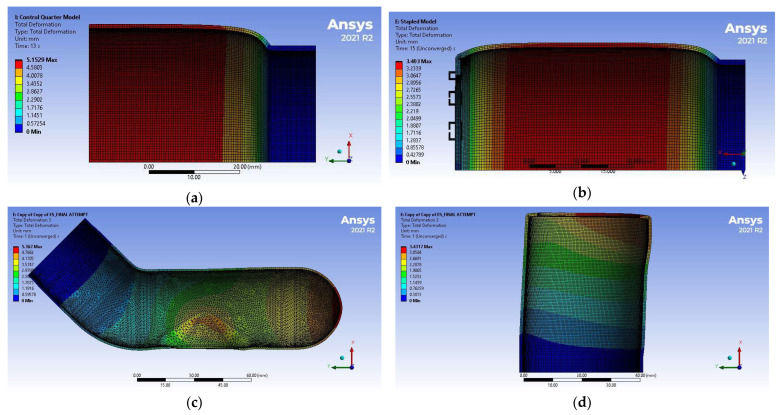
Displacement distribution of the FEM simulations: (**a**) Control, (**b**) EE, (**c**) ES proximal end, (**d**) ES distal end. Models are not shown on the same scale.

**Figure 17:**
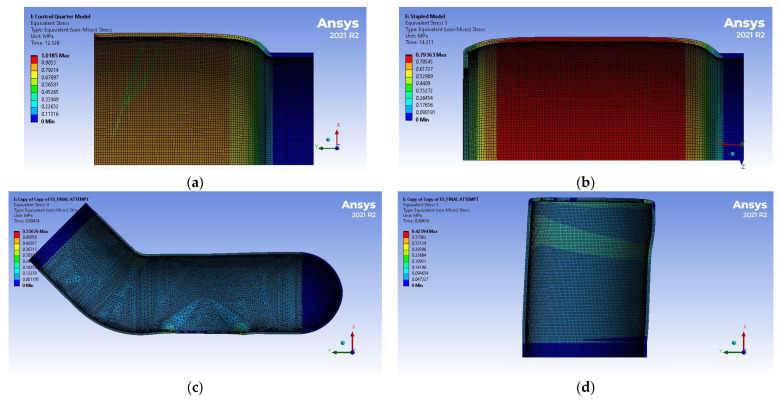
Stress distribution of the FEM simulations: (**a**) Control, (**b**) EE, (**c**) ES proximal end, (**d**) ES distal end. Models are not shown on the same scale.

**Figure 18:**
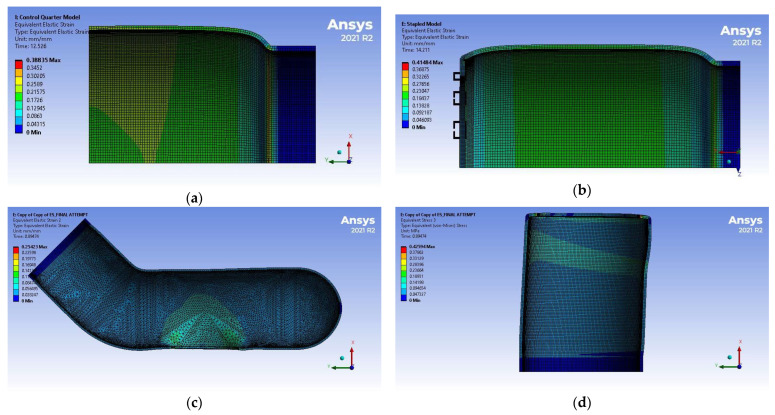
Strain distribution of the FEM simulations: (**a**) Control, (**b**) EE, (**c**) ES proximal end, (**d**) ES distal end. Models are not shown on the same scale.

Table 9 and Figure 16 summarize the displacement results of the simulations. The maximum diameter corresponds to the average displacement of the elements along the section with the biggest expansion. Stress and strain distributions are shown in Figure 17 and Figure 18, respectively.

**Figure 19:**
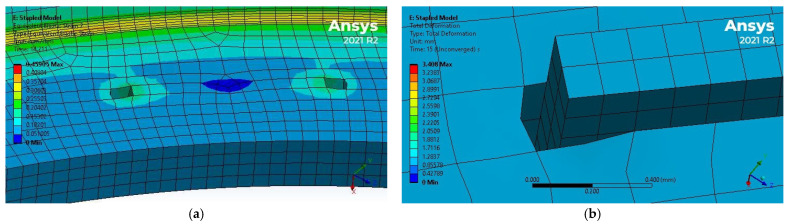
Interaction between staples and colorectal tissues in EE simulation: (**a**) low-strain region of the tissues within the staple, (**b**) internal pressure caused the expansion of the staple puncture hole. The figures are not shown on the same scale.

As expected, the colorectal tissues between the staple legs experienced limited strains and stresses as the staples are much stiffer than the tissue. Additionally, it was noted that the staple puncture holes experienced elongation along the circumferential direction, which may increase the chances of leakage; see Figure 19.

## 4. Discussion

### 4.1. Experimental Results Discussion

As Table 4 shows, AL primarily occurred at the circular staple lines as opposed to linear staple lines or tissues for both EE and ES anastomoses, which is consistent with clinical observations. The specimens that experienced AL along the circular staple lines were compared to Control specimens with tissue leakage; see Table 5. These results show that while the thicknesses and the diameters of the colonic tissues used in these experiments were of the same order, the diameter at AL varied with the biggest diameter occurring at Control specimens while EE and ES experienced smaller diameters at leakage. Based on Table 5, the standard deviation as a percentage of the average of Control, EE, and ES are 46%, 26%, and 48%, respectively. This shows that Control and ES specimens were behaving closely, which would make ES a preferable choice. Investigating the reasons for the higher standard deviation in ES should be the topic of a follow-up study.

The standard deviation of the Control specimens is significantly higher than those of EE and ES specimens. Interestingly, the results of Table 5 show nearly identical leak pressure of both EE and ES specimens, which is slightly lower than that of the Control specimens. These results are comparable to those reported by other researchers, e.g., [37], where trans-anal minimally invasive surgeries and endoscopic sutures have about the same leak pressure. Time to leakage varied a lot between the three groups, with the EE specimen having the shortest time to leakage, while ES specimens took more time to leak than Control specimens. Time to leakage of ES was significantly higher than with EE with an unpaired *t*-test with *p* < 0.05, even though time to leakage had a much higher standard deviation for the ES specimens than the EE specimens. Leak pressure of EE and ES specimens had lower standard deviations than the intact Control specimen segments. On the other hand, the time to leakage of EE specimens had the lowest standard deviation of the three groups. The standard deviation is the highest with the Control specimens. These results were confirmed by multiple comparisons of the EE and ES groups versus the Control group, which showed that, except for the initial diameter, EE and ES have means that are significantly different than that of the Control group.

These observations are reasonable since the ability of the colonic section to stretch was limited by introducing the staplers, especially in the case of EE, which did not have a pouch to allow stretching. This led to higher stress in the tissues and, consequently, earlier failure. Colonic tissues are hyperelastic, with fiber bundles of varying strengths that are distributed along different orientations, which leads to a wide distribution of colonic section strengths as reported by [30,38,39]. Inflating tissues until leakage in intact Control specimens resulted in a bigger variation in diameter at leakage, leak pressure, and time to leakage. Staples in the anastomosis introduced a ‘weak link,’ reducing diameter at leakage and leak pressure. Anastomosis made leakage more consistent and therefore reduced the standard deviations of these two parameters. Similar observations were reported in [35]. Interestingly, the structure of ES led to a significantly longer time to leakage. The added volume in the pouch probably contributed to this phenomenon.

The observations discussed above were confirmed by the results shown in Table 7, where the three groups are more distinct than in raw data with ∆σ of ES close to that of the Control while EE has the lowest ∆σ. The same can be observed for as ϵ, where ES allows for greater expansion of the colon while EE has a more restrictive nature. The standard deviation observation described above is consistent with what can be observed for ∆σ, where the Control had the highest standard deviation while ϵ of the Control had the lowest standard deviation. The results may explain why several clinical studies have shown ES to have a lower leak rate than ES [18]. Colorectal tissue is extremely dynamic in humans. The stress and strain on the tissue is a function of intraabdominal pressure, tissue edema and overall patient volume status, composition of feculent load, and a multitude of other physiologic factors. Anastomoses that can accommodate strain for a longer time without failure are less likely to result in a clinically significant leak.

### 4.2. Discussion of FEM Results

Developing a model of any physical system requires several assumptions. In this work, the main assumptions were modeling the colorectal sections as cylinders and simplifying the geometry of the staples.

As described in Section 2.6, the meshes of a staple and the surrounding tissues originally coincided. As the internal pressure increases, the resulting tensile stress in the tissues tends to expand the tissues. However, this process is not uniform since the tissues adjacent to the inner side of the staple are restricted by the staple while those adjacent to the outer side of the staple are relatively less obstructed. As the pressure increases during simulation, the tissues on the outer surface of the staple will separate from the staple, indicating the onset of leakage.

The accuracy of the model depends on the deviation of the assumption from the actual system and the closeness of the experimental results to those of the model. Overall, the FEM studies correlated very well with the observed ex vivo results. In all simulations, the maximum diameter was within the range of diameter at leakage. The percentage of FEM errors with respect to the average diameter of leakage amounted to 4%, 3%, and 8% for Control, EE, and ES specimens, respectively.

## 5. Conclusions

This research develops a reproducible ex vivo experimental setup to study the integrity of colorectal anastomoses immediately after surgeries using an air pressure leakage test, which is typically one of the final steps of these surgeries. This setup was used to evaluate two common colorectal anastomosis techniques: end-to-side and end-to-end, compared to Control specimens. Porcine colorectal tissues were used in these experiments. It was found that EE and ES have nearly identical leak pressures, which were slightly lower than the leak pressures of the Control specimens. However, ES anastomoses have a longer time to leakage and a bigger diameter at leakage than EE anastomoses, which were probably caused by the addition of the ES pouch. Therefore, ES anastomoses can better accommodate internal pressure compared to EE anastomoses. The experimental results were further clarified by introducing two variables: ∆σ and ϵ, to account for the variation in diameter of the colonic tissues. As Section 4 shows, using these two variables helped show the advantage of ES over EE. This research may help explain the reduced risk of anastomotic leak after low anterior resection for ES versus the EE anastomoses that was reported in prior in vivo studies (e.g., [18]). An approach to simulate EE and ES anastomoses using FEM was also proposed. These simulations considered the effects of the hyperelastic colonic tissues and the interaction between the staples and the tissues. The simulation and experimental results matched well.

Potential next steps of this research may include testing additional colorectal anastomosis types and new stapler technologies. Simulations can be further developed to predict AL more precisely and to compare various types of staples and optimize the stapling arrangements before surgical implementation. A probabilistic FEM study can better account for the uncertainty in factors such as loading conditions, material behavior, and geometric configurations. Such a study will provide rational reliability analysis and describe the behavior of an anastomosis. Surgical staplers are expensive, some costing over USD 10,000 for a single use. Using FEM studies to identify promising staple configurations and confirming these results with much fewer ex vivo and in vivo studies may allow for superior stapler device designs at much lower cost. Additionally, with enhanced recovery after surgery protocols being implemented internationally and rapid return of bowel function after surgery, being able to reproducibly model colorectal anastomoses put under immediate stress and strain with ex vivo and FEM modeling techniques is more relevant than ever. A future follow-up in vivo study in a porcine model can help assess the differences in healed anastomotic site strength post-healing. Such research can further elucidate the differences between EE and ES techniques in terms of long-term healing.

Combined FEM simulation and ex vivo experimental testing may lead to a better understanding of AL and the design of new anastomotic configurations with higher mechanical strength and potentially lower leak rates.

## Figures and Tables

**Table 1:** Nickle–titanium alloy bilinear model parameters.

Yield Strength (MPa)	Poisson’s Ratio	Modulus of Elasticity (GPa)	Tangent Modulus (MPa)
930	0.3	96	2150

**Table 2:** Element sizes and corresponding element counts used for Control, EE, and ES models.

Model	Meshing Technique	Element Type	Element Size (mm)	Element Count
** Control **	Multi-zone	Hexahedral	0.5	16,362
** EE **	Multi-zone	Hexahedral-Dominant Hexahedral-Dominant Hexahedral	0.5 (Tissues, general) 0.5 (Tissues, around staples) 0.125 (Staples)	36,035
** ES **	Patch Conforming Method	Tetrahedral (Proximal End) Tetrahedral (Proximal End) Hexahedral	1.0 (Proximal End) 0.5 (Distal End) 0.125 (Staples)	98,434

**Table 3:** Coefficients of the anisotropic hyperelastic constitutive porcine colorectal tissue model.

***a***_**1**_ (Pa)	***a***_**2**_ (Pa)	***a***_**3**_ (Pa)	
1.400 × 10^5^	−9.585 × 10^3^	−1.976 × 10^3^	
***c***_**2**_ **(Pa)**	***c***_**3**_ **(Pa)**	***c***_**4**_ **(Pa)**	***β***_**1**_ **(Degrees)**
4.929 × 10^5^	−2.981 × 10^5^	6.553 × 10^3^	0.790
***e***_**2**_ **(Pa)**	***e***_**3**_ **(Pa)**	***e***_**4**_ **(Pa)**	***β***_**2**_ **(Degrees)**
3.0146 × 10^4^	−2.9556 × 10^4^	−739.324	90.100

**Table 4:** Summary of the leakage testing.

	Control	EE	ES
Tissue failure	22	2	1
Slippage	0	0	2
Corrupt/low quality video	8	11	3
Linear staple leakage	N/A	1	3
Circular staple leakage	N/A	16	21

The grey cells corresponds to *n for each case.*

**Table 5:** Comparison of EE and ES specimens that failed along the circular staple line and Control specimens that experienced tissue failure.

	Control (*n* = 22)	EE (*n* = 16)	ES (*n* = 21)
Pre-testing thickness (mm)	1.0 ± 0.1	1.0 ± 0.1	1.0 ± 0.1
Leak pressure (mm Hg)	294.4 ± 12.1	282.6 ± 3.0	282.8 ± 2.6
Initial diameter (mm)	43.6 ± 8.1	42.2 ± 6.5	40.1 ± 5.7
Diameter at leakage (mm)	56.3 ± 8.4	50.5 ± 7.1	52.8 ± 6.5
Time to leakage (s)	194.5 ± 90.2	106.3 ± 28.1	263.9 ± 127.0

**Table 6:** Variance comparison of EE and ES specimens that failed along the circular staple line to Control specimens that experienced tissue failure.

		* p * -Value	Confidence Interval	
			Lower	Upper
Initial diameter	EE vs. Control	0.387	0.566	3.931
	ES vs. Control	0.125	0.819	4.862
Diameter at leakage	EE vs. Control	0.560	0.492	3.416
	ES vs. Control	0.255	0.684	4.058
Time to leakage (s)	EE vs. Control	1.160 × 10^−13^	57.109	396.518
	ES vs. Control	0.127	0.206	1.221

**Table 7:** Multiple comparison of **Control**, EE, and ES specimens that failed along the circular staple line to Control specimens that experienced tissue failure.

	Group	Control Group	Lower Limit	Difference	Upper Limit	* p * -Value
** Leak pressure **	EE	Control	−206.279	−197.941	−189.603	1.110 × 10^−16^
	ES	Control	−19.366	−11.624	−3.882	0.002
** Initial diameter **	EE	Control	−6.678	−1.369	3.941	0.787
	ES	Control	−8.387	−3.458	1.477	0.204
** Diameter at leakage **	EE	Control	−11.499	−5.82	−0.134	0.044
	ES	Control	−8.756	−3.479	1.797	0.242
** Time to leakage **	EE	Control	10.348	21.372	32.397	9.415 × 10^−5^
	ES	Control	15.744	25.980	36.216	7.082 × 10^−7^

**Table 8:** ∆σ and ϵ for EE, ES, and Control results.

	Control (*n* = 22)	EE (*n* = 16)	ES (*n* = 21)
∆σ (mm Hg)	374 ± 15	235 ± 2	359 ± 3
∆σ (MPa)	0.0498 ± 0.0020	0.0313 ± 0.0003	0.0479 ± 0.0004
ϵ	0.29 ± 0.04	0.20 ± 0.09	0.32 ± 0.14

**Table 9:** Comparison of the displacement results for the three simulation models.

	Control	EE	ES, Proximal End
Initial diameter (mm)	43.6	42.2	40.1
Average maximum diameter (mm)	53.8	49.0	48.7

## Data Availability

The datasets presented in this article are not readily available as the data are part of an ongoing study. Requests to access the datasets should be directed to the corresponding author.

## Abbreviations

The following abbreviations are used in this manuscript:

AL	Anastomotic leakage
EE	End-to-end colorectal anastomosis
ES	End-to-side colorectal anastomosis
FEM	Finite element method
